# Transjugular Intrahepatic Portosystemic Shunt Placement in Patients with Schistosomiasis-Induced Liver Fibrosis

**DOI:** 10.1007/s00270-019-02295-6

**Published:** 2019-07-30

**Authors:** Jiacheng Liu, Binqian Zhou, Dongpin Chen, Chen Zhou, Qin Shi, Chuansheng Zheng, Gansheng Feng, Feng Yuan, Yan Ge, Bin Xiong

**Affiliations:** 1 Department of Radiology, Union Hospital, Tongji Medical College, Huazhong University of Science and Technology, Jiefang Avenue #1277, Wuhan, 430022 China; 2 Department of Ultrasound, Union Hospital, Tongji Medical College, Huazhong University of Science and Technology, Wuhan, 430022 China; 3 Hubei Province Key Laboratory of Molecular Imaging, Wuhan, 430022 China

**Keywords:** Schistosomiasis, Transjugular intrahepatic portosystemic shunt, Portal hypertension, Gastroesophageal variceal bleeding, Hepatic encephalopathy

## Abstract

**Purpose:**

Evaluate the efficacy and safety of transjugular intrahepatic portosystemic shunt (TIPS) insertion on patients with schistosomiasis-induced liver fibrosis, and compare with that of patients with HBV-induced cirrhosis.

**Materials and Methods:**

This was a retrospective study from November 2015 to December 2018 including 82 patients diagnosed with portal hypertension, one group of which is induced by schistosomiasis (*n* = 20), the other by hepatitis B virus (HBV) (*n* = 62). Both groups of subjects underwent TIPS placement for the management of portal hypertension complications.

**Results:**

TIPS was inserted successfully in all patients (technical success 100%). After a median follow-up of 14 months following TIPS insertion, portal pressure gradient (PPG) value in both schistosomiasis-induced group and HBV-induced group underwent a significant decrease with no major difference between the two groups. There exists no significant difference demonstrated by Kaplan–Meier curves between two groups concerning cumulative rate of hepatic encephalopathy (HE) (log-rank *p* = 0.681), variceal rebleeding (log-rank *p* = 0.837) and survival (log-rank *p* = 0.429), and no statistically difference was found in terms of alleviation of portal vein thrombosis (PVT). In addition, splenectomy (HR 19, 95% CI 4–90, *p* < 0.001) was identified as independent predictor of PVT.

**Conclusions:**

TIPS placement is well-founded to be considered as a safe and effective treatment in patients with schistosomiasis-induced portal hypertension and relevant severe complications. We also found the risk of PVT is 19 times higher in patients who underwent splenectomy than in untreated patients.

**Level of Evidence:**

Historically controlled studies, level 4.

## Introduction

Schistosomiasis, a generally incurable and highly infectious disease, has gained stepwise attention over the decades. Although mass drug administration (MDA) programs have been used for many years, there are still 240 million people being affected with schistosomiasis worldwide currently [1, 2]. Schistosomiasis is a snail-borne disease caused by trematodic worms of genus schistosoma, which includes three main species, *Schistosoma haematobium*, *Schistosoma mansoni* and *Schistosoma japonicum* [3, 4]. *Schistosoma haematobium* causes urogenital schistosomiasis, and other species cause intestinal schistosomiasis. In China, the most common one is *schistosoma japonicum*, which occurs mainly in the Yangtze River valley especially in Hubei Province [5–7].

The major pathology of schistosomiasis is associated with the occurrence and development of tissue fibrosis. In the case of *S. mansoni* and *S. japonicum* infections, as the eggs accumulate in the terminal portal branches, causing periportal fibrosis, the resulting obstruction can lead to several complications associated with presinusoidal portal hypertension, such as ascites and gastroesophageal varices bleeding, which can be fatal in case of severe variceal hemorrhage [3].

Transjugular intrahepatic portosystemic shunt (TIPS) intervention is a critical technique for patients with refractory ascites and recurrent variceal bleeding secondary to portal hypertension, which patients with schistosomiasis-induced liver fibrosis are more likely and will ultimately suffer from. Schistosomiasis-induced portal hypertension, characterized by portal fibrosis, has always been identified as non-cirrhotic portal hypertension. There are still doubts about the propriety of TIPS implantation for it is rather difficult [8]. According to current consensus conferences and guidelines, it is not literally listed as an indication for TIPS [9–11]. One can only retrieve six cases reported worldwide on the application of TIPS on schistosomiasis-induced liver fibrosis [12–15].

Here, we report the application of TIPS for the management of patients with schistosomiasis-induced liver fibrosis and cirrhosis attributed to hepatitis B virus (HBV), compare the therapeutic outcome between these two groups and thereby analyze the possible parallel efficacy of TIPS treatment on both schistosomiasis-induced liver fibrosis and HBV-induced cirrhosis.

## Materials and Methods

A retrospective analysis was performed in our center ranging from November, 2015 to December, 2018. During this period, 237 patients underwent TIPS, with only 82 meet the inclusion criteria. The study was performed in accordance with the principles of good clinical practice, the principles of the declaration of Helsinki and its appendices and local and national laws. Written informed consent was obtained for all patients.

### Patient Selection

The inclusion criteria were refractory ascites and recurrent gastroesophageal variceal bleeding resulted from schistosomiasis or HBV, and there were no cerebral invasion in patients with schistosomiasis. The exclusion criteria were patients complicated with contraindications of TIPS, hepatorenal syndrome or hepatic encephalopathy. In accordance to Baveno VI [9] and American Association for the Study of Liver Diseases (AASLD) criteria [10], recurrent gastroesophageal variceal bleeding is defined as ineffectiveness of applying first-line therapy [non-selective beta blocker (NSBB) + endoscopic variceal ligation (EVL)] to prevent rebleeding. Refractory ascites is defined as if it could not be mobilized or recurred early after paracentesis and could not be prevented by sodium restriction and diuretic treatment [16].

**Fig. 1:**
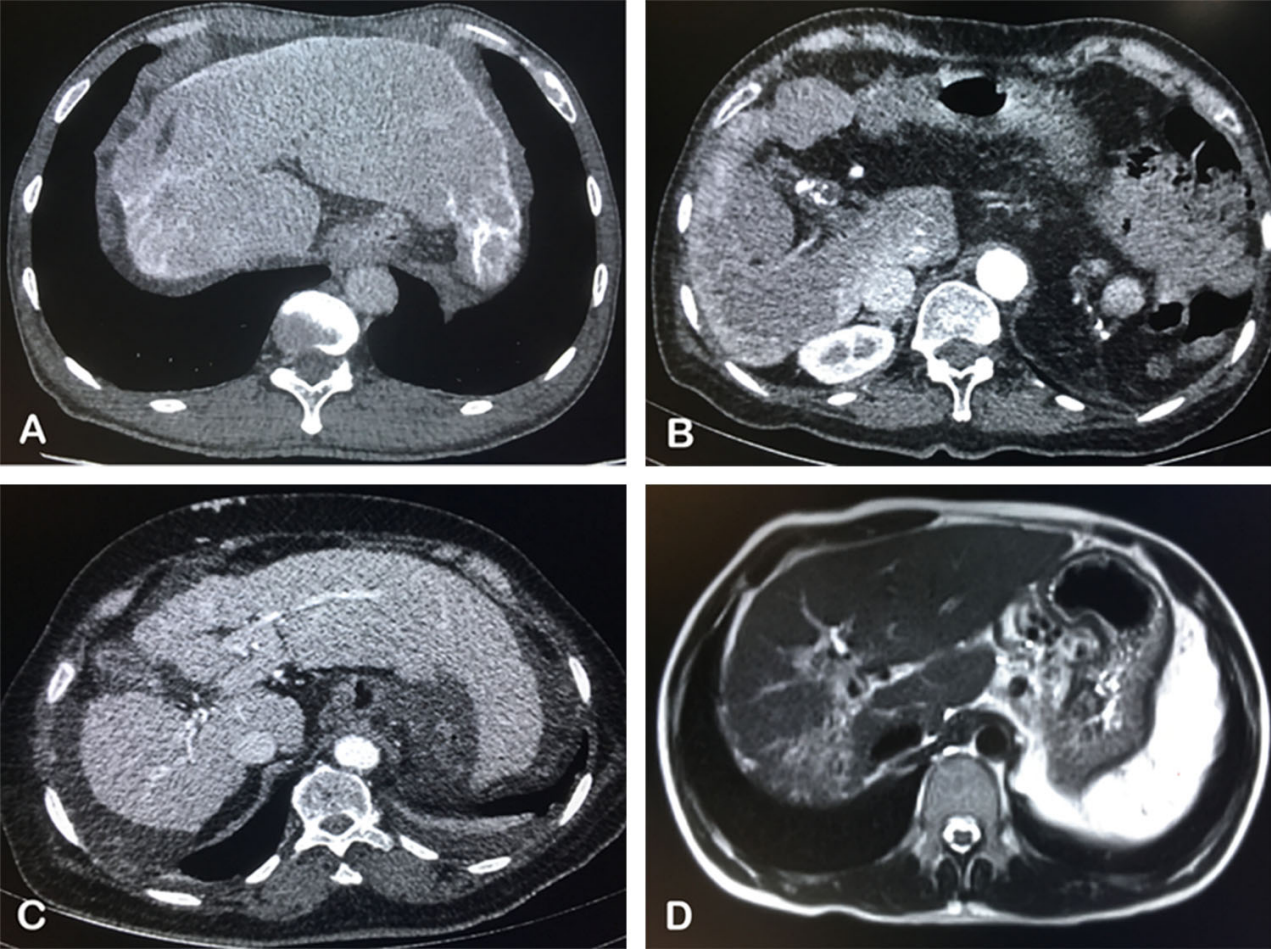
Typical symptoms of schistosomiasis-induced liver fibrosis. **A** The capsular calcification was found in the CT scan of a 52-year-old man; **B** widening of ligamentum teres, portal vein thrombosis (PVT) and insufficient liver perfusion in the CT enhanced scan of a 54-year-old woman; **C** ascites and prominent collateral circulation in the CT enhanced scan of a 70-year-old woman; **D** portal branches fibrosis in the T2-weighted image of a 31-year-old man

After ruling out unqualified 155 patients based on exclusion criteria, 20 subjects with schistosomiasis-induced liver fibrosis and 62 with HBV-induced cirrhosis were included. The diagnostic criteria of advanced schistosomiasis have been described before [17]. Twenty schistosomiasis-induced patients went through the routine imaging examinations including ultrasonography, computed tomography (CT) scan and endoscopy before TIPS insertion, and typical symptoms were found in all [18–21], including portal branches fibrosis, capsular calcification of liver, ascites and prominent collateral circulation (Fig. 1).

Of the 20 patients in the schistosomiasis-induced group, recurrent gastroesophageal varices bleeding occurred in 16 and refractory ascites occurred in 4. And 9 of them had received endoscopic variceal ligation (EVL), 12 had received splenectomy and azygoportal disconnection before TIPS procedure, but none of curative effect was observed. As for the 62 patients in the HBV-induced group, indication for TIPS in 7 patients was refractory ascites, and in the other 55 patients it was gastroesophageal varices bleeding. The baseline characteristics of both groups are depicted in Table 1.

**Table 1:** Baseline characteristics of patients included in the study

Variables	Schistosomiasis (*n* = 20)	HBV (*n* = 62)	*p* values
Age (years)	56.4 ± 12.6	50.1 ± 10.6	0.276
Sex (male)	14 (70.0%)	50 (80.6%)	0.358
Emergency bleeding	5 (25.0%)	21 (33.9%)	0.585
History of hypertension and(or) diabetes	5 (25.0%)	12 (19.4%)	0.752
History of hepatic carcinoma	1 (5.0%)	6 (9.7%)	0.453
History of splenectomy	12 (60.0%)	9 (14.5%)	**< 0.001**
TIPS indication			0.449
Recurrent gastroesophageal variceal bleeding	16 (80.0%)	55 (88.7%)	
Refractory ascites	4 (20.0%)	7 (11.3%)	
Laboratory parameters			
Total bilirubin (umol/L)	19.5 ± 9.2	30.1 ± 46.2	0.314
Albumin (g/L)	30.3 ± 5.3	31.0 ± 5.9	0.698
Alanine aminotransferase (U/L)	41.0 ± 32.8	35.4 ± 36.3	0.547
Aspartate aminotransferase (U/L)	52.5 ± 40.6	43.2 ± 34.3	0.334
Creatinine (umol/L)	120.3 ± 204.5	68.3 ± 21.8	0.271
Blood urea nitrogen (mmol/L)	6.74 ± 4.12	6.10 ± 2.45	0.418
Prothrombin time (s)	16.7 ± 4.3	17.2 ± 2.4	0.522
International normalized ratio	1.37 ± 0.47	1.43 ± 0.25	0.542
Hemoglobin (g/L)	78.6 ± 14.4	74.9 ± 20.7	0.380
Platelet count (10^9^/L)	170.1 ± 132.1	75.5 ± 54.9	**0.005**
Serum Na (mmol/L)	139.1 ± 3.7	139.0 ± 4.1	0.915
Child–Pugh score	7.9 ± 1.3	7.7 ± 1.6	0.555
MELD score^a^	11.7 ± 4.9	11.9 ± 3.8	0.878
MELD-Na score	12.1 ± 4.8	12.5 ± 4.6	0.724
Imaging evaluation			
Portal vein diameter (mm)	14.5 ± 3.8	15.8 ± 3.5	0.675
Gastric coronary vein diameter (mm)	6.6 ± 2.7	6.5 ± 3.0	0.855
Splenic vein diameter (mm)	11.7 ± 4.4	11.9 ± 3.0	0.872
Spleen diameter (cm)	15.4 ± 2.6	16.6 ± 3.1	0.286
PVT score^b^	1.6 ± 1.9	0.6 ± 0.9	**< 0.001**
Pre-TIPS PPG (mmHg)	26.4 ± 4.9	27.7 ± 4.5	0.277
Pre-TIPS portal pressure (mmHg)	33.3 ± 5.4	34.4 ± 5.0	0.404
Duration of follow-up (months)	14.4 ± 8.4	15.0 ± 7.3	0.293

Bold values indicate statistical significance

^a^*MELD* model of end-stage liver disease [39]

^b^*PVT* Portal vein thrombosis, the PVT location consists of the main portal vein (MPV), superior mesenteric vein (SMV) and splenic vein (SV). Whose severity was divided into four levels: grade 0 (no thrombosis), grade I (MPV thrombus < 50% or only SMV and SV thrombus existed), grade II (MPV thrombus accounted for 50–100%) and grade III (complete blocking or cavernous transformation of the portal vein) [40, 41]. Four levels were each scored 0/1/2/3

### TIPS Procedure

**Fig. 2:**
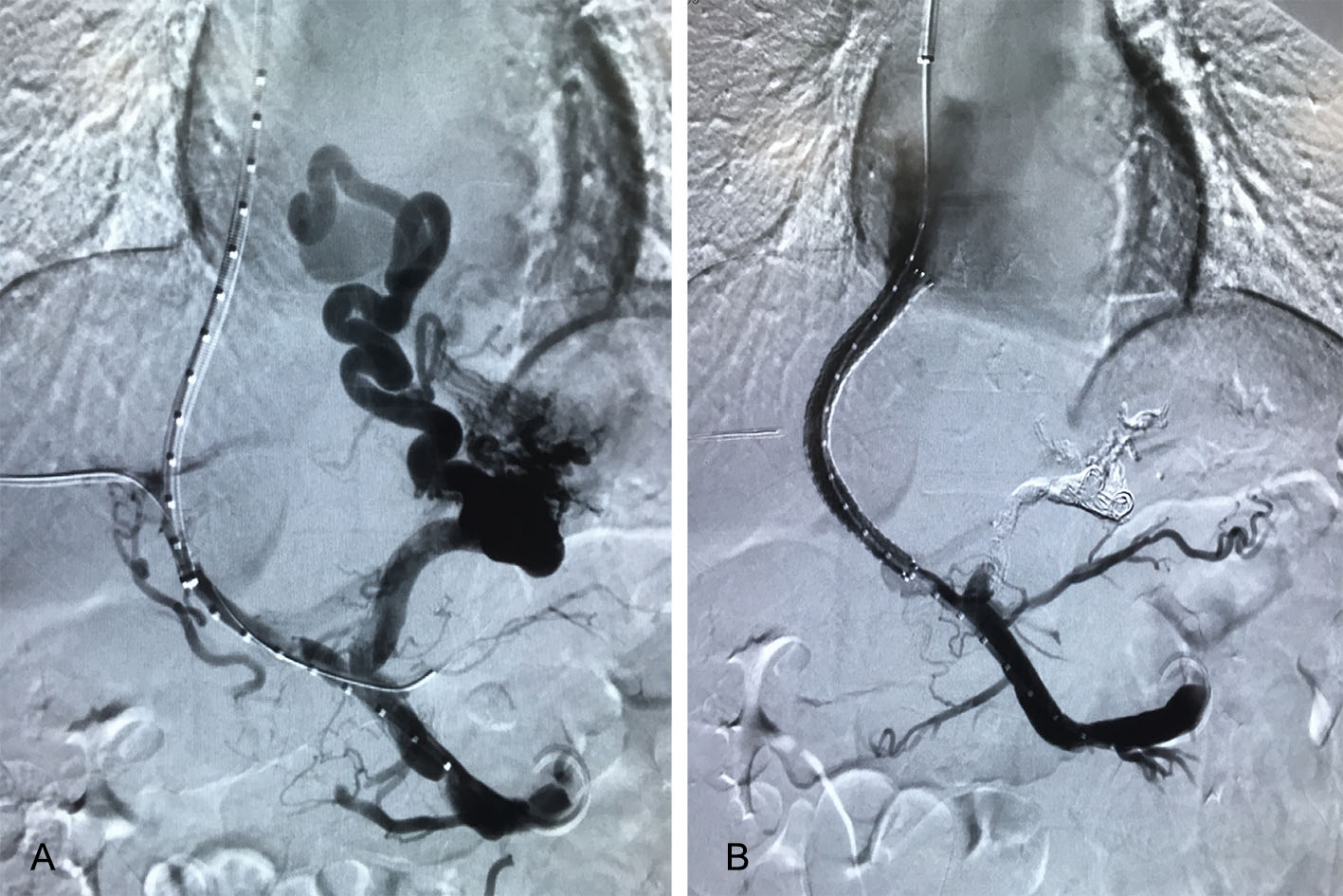
The images of TIPS procedure on a 31-year-old male with schistosomiasis-induced portal hypertension. **A** Portal vein was successfully punctured, and pigtail catheter was placed in SMV, followed by angiography revealing PVT and gastric esophageal varices; **B** after TIPS insertion, blood perfusion was satisfying in the stent as displayed

TIPS procedure has been depicted in detail before [22]. In our study, the procedure was operated according to the instructions by an interventional radiologist with ten-year experience. Briefly, a bare metal stent (Bard E-LUMINEXX, Vascular Stent, Karlsruhe, Germany) combined with 8-mm expandable PTFE-covered stent (Fluency, Bard Peripheral Vascular, Tempe, AZ, USA) was inserted in the entire length of the intrahepatic tract, which were initially dilated to 6 mm. If the PPG remained more than 12 mmHg, further stent dilatation up to 7 mm or 8 mm was considered. Hepatic venous pressure gradient and portal vein pressure were measured before and after the stent placement. Besides, a mixture of bucrylate (Histoacryl, B. Braun Surgical, Rubi, Spain) and Lipiodol (Lipiodol^®^ Ultra-Fluid, Guerbet, Roissy, France) (1:1–1:3 vol/vol) was utilized to embolize gastric varices once found via angiography (Fig. 2).

After the procedure, all patients were required to stay in hospital for several days and received symptomatic treatments, such as analgesia, antipyretic and anticoagulation. In patients with PVT, warfarin was administered orally for 6 months or until the portal vein system was completely recanalized. If portal vein system is recanalized shortly, warfarin was withdrawn after 6 months; if not or just partially recanalized, anticoagulation treatment was maintained more than 6 months. The dose of warfarin was administered to reach the target international normalized ratio of 2–3. If potential thrombosis is found, long-term warfarin should be prescribed [23]. Lactulose was routinely administered in all patients after TIPS to induce 1–2 loose stools per day. Diuretic drugs were dispensed as necessary. Moreover, appropriate doses of praziquantel were needed for control of any progress of disease in schistosomiasis-induced group.

### Follow-Up and Observation Index

At 3, 6, 12, 18, 24 months after the TIPS procedure, follow-up visits were scheduled for every individual. During the follow-up, laboratory tests and imaging examinations such as Doppler ultrasonography or CT scan were carried out. Laboratory tests mainly included liver, kidney and coagulation function. The study outcomes were the incidence of hepatic encephalopathy (HE) [24], variceal rebleeding, survival as well as shunt dysfunction which was defined as shunt thrombosis or stenosis resulting in clinical symptoms and/or requiring procedural intervention [25].

### Statistical Analyses

Statistical analyses were performed using SPSS software version 22.0, and *p* values < 0.05 were considered statistically significant. Continuous variables are presented as the mean ± standard deviation and were compared by independent sample t tests or paired-sample *t* tests accordingly. Categorical data were expressed as absolute and percentage values and compared using *χ*^2^ test and Mann–Whitney *U* tests, respectively. Time-to-event outcomes were performed by the Kaplan–Meier method and compared by log-rank test. Cox regression model was used to identify independent predictors.

## Results

TIPS was inserted successfully in all patients (technical success 100%). Of the 16 patients with gastroesophageal varices hemorrhage in the schistosomiasis-induced group, 7 were esophageal varices (EV) and 9 were gastric varices (GV) (according to Sarin’s classification [26], 8 were classified as GOV1 and 1 as IGV2). Of the 55 patients with gastroesophageal varices hemorrhage in the HBV-induced group, 20 were EV and 35 were GV (28 were classified as GOV1 and 4 as GOV2, 2 as IGV1, 1 as IGV2). During the TIPS procedure, bucrylate was utilized to embolize GV once found.

We found difference between these two groups concerning splenectomy, platelet count and PVT, which is on the account that splenectomy and azygoportal disconnection is the current dominant treatment for schistosomiasis-induced liver fibrosis in China, and the average platelet count is therefore higher than HBV-induced group. However, it has no effect on comparing the therapeutic outcome and complications of TIPS between two groups of patients, for no difference is discovered on other baseline characteristics such as pre-TIPS PPG, hepatic function, etc. In terms of the occurrence of PVT before TIPS insertion, the univariate analysis showed that history of schistosoma (*p* = 0.038), splenectomy (*p* < 0.001) and higher platelet count (*p* = 0.001) are related. The multivariate analysis showed that only splenectomy (HR 19, 95% CI 4–90, *p* < 0.001) was identified as independent predictors of PVT (Table 2).

**Table 2:** Predictors of PVT before TIPS placement in the time-to-event analysis

	PVT (*n* = 39)	No PVT (*n* = 43)	*p* value
Sex (male)	33 (84.6%)	31 (72.1%)	0.193
Age (years)	51.2 ± 11.1	52.1 ± 11.7	0.712
Confirmed schistosoma patients	14 (35.9%)	6 (14.0%)	**0.038**
History of hypertension and(or) diabetes	3 (15.4%)	4 (25.6%)	0.288
History of hepatic carcinoma	2 (7.7%)	5 (9.3%)	0.555
History of splenectomy	19 (48.7%)	2 (4.7%)	**< 0.001**
Laboratory parameters			
Total bilirubin (µmol/L)	33.1 ± 55.1	21.7 ± 13.4	0.220
Albumin (g/L)	29.9 ± 5.7	31.7 ± 5.6	0.153
Alanine aminotransferase (U/L)	41.0 ± 41.9	32.9 ± 27.3	0.320
Aspartate aminotransferase (U/L)	50.6 ± 38.6	41.0 ± 33.2	0.249
Creatinine (µmol/L)	90.9 ± 147.3	73.7 ± 44.5	0.493
Blood urea nitrogen (mmol/L)	6.49 ± 3.41	6.07 ± 2.50	0.544
Prothrombin time (s)	16.9 ± 2.7	17.2 ± 3.2	0.576
International normalized ratio	1.39 ± 0.29	1.44 ± 0.35	0.528
Hemoglobin (g/L)	75.6 ± 18.7	79.6 ± 13.3	0.281
Platelet count (10^9^/L)	134.8 ± 114.2	66.5 ± 40.2	**0.001**
Serum Na (mmol/L)	138.9 ± 4.2	139.2 ± 3.9	0.759
Child–Pugh score	7.9 ± 1.6	7.5 ± 1.5	0.215
MELD score	11.8 ± 4.5	11.8 ± 3.7	0.968
MELD-Na score	12.4 ± 5.0	12.4 ± 4.3	0.995
Imaging evaluation			
Portal vein diameter (mm)	15.6 ± 4.3	15.7 ± 3.7	0.905
Gastric coronary vein diameter (mm)	6.4 ± 3.3	6.6 ± 2.6	0.754
Splenic vein diameter (mm)	12.1 ± 3.7	11.8 ± 2.8	0.714
Spleen diameter (cm)	16.1 ± 3.1	16.6 ± 3.0	0.540
Pre-TIPS PPG (cmH_2_O)	36.9 ± 6.2	37.6 ± 6.2	0.614
Pre-TIPS portal pressure (cmH_2_O)	46.9 ± 7.9	45.7 ± 5.8	0.443

	HR	95% CI	*p* value
Multivariate analysis			
Confirmed schistosoma patients	–	–	–
History of splenectomy	19.000	4.002–90.203	< 0.001
Platelet count (10^9^/L)	–	–	–

Bold values indicate statistical significance

TIPS procedure was conducted successfully on all 82 patients, the median follow-up times for the schistosomiasis-induced and HBV-induced groups were 15 (2–30) and 15 (2–27) months. After TIPS placement, The portal pressure gradient (PPG) of all the patients fell from 27.4 ± 4.6 mmHg to 10.2 ± 3.4 mmHg, *p* < 0.001, 73 patients (89.0%) even to below 12 mmHg. Conclusively, they all had a decrease of at least 20% from baseline. During the follow-up, variceal rebleeding or first variceal hemorrhage (for patients with refractory ascites as TIPS indicator) was found in 12 patients (14.6%), HE in 19 (23.2%), 9 (11.0%) deaths, with none shunt dysfunction.

### Comparison of PPG Dropping Degree After TIPS Placement

ΔPPG is defined as reduction in PPG values after TIPS placement. The average ΔPPG of schistosomiasis-induced group is 16.4 ± 3.9 mmHg, and the HBV-induced group is 17.5 ± 4.0 mmHg, *p* = 0.314; the descending ratio of PPG after TIPS intervention (ΔPPG/pre-TIPS PPG*100%) is averaged 62.6% ± 7.1% in former, 62.7% ± 8.8% in latter, *p* = 0.978. In addition, post-PPG below 12 mmHg is found in 18 schistosomiasis-induced patients (90.0%), 55 HBV-induced patients (88.7%), *p* = 0.619. Thus, we can draw the conclusion that the efficacy of TIPS on reducing PPG is undifferentiated between schistosomiasis-induced and HBV-induced portal hypertension.

### Comparison of Clinical Outcomes After TIPS Placement

#### PVT Outcome

There are 39 patients being diagnosed with PVT when enrolling, including 14 in the schistosomiasis-induced group and 25 in the HBV-induced group. The recanalization of PVT after TIPS placement was considered complete if enhanced CT showed the complete absence of filling defects in the MPV, SMV and SV. Recanalization was considered partial if it achieved a decrease in severity of PVT in at least one vein [27], any other condition is considered not alleviated. Efficacious therapy is defined as complete or partial recanalization of portal vein system thrombosis. In the schistosomiasis-induced group, after TIPS insertion, 4 (28.6%) of them were considered complete recanalization, 5 (35.7%) were partial recanalization, and 5 (35.7%) were not alleviated. In the HBV-induced group, after TIPS insertion, 8 (32.0%) of them were considered complete recanalization, 8 (32.0%) were partial recanalization, and 9 (36.0%) were not alleviated, *p* = 0.965. Also, these two groups do not differ from each other concerning the effectiveness of TIPS on PVT.

#### HE

**Fig. 3:**
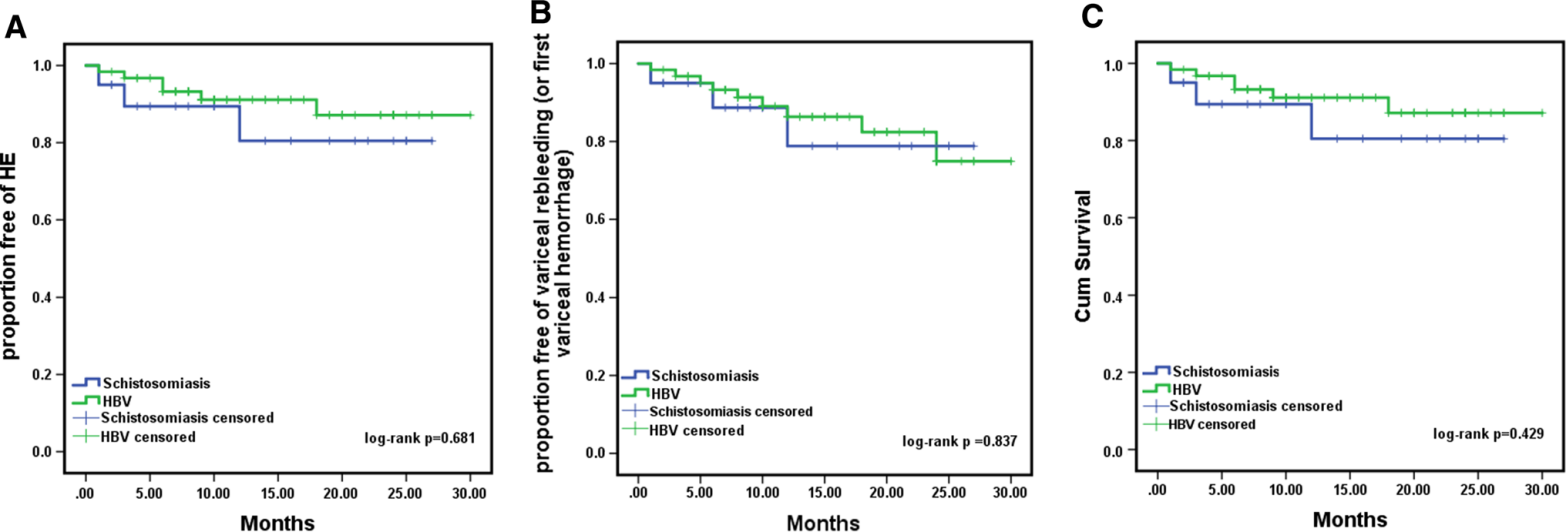
Kaplan–Meier curves of HE (**A**), rebleeding (**B**) and cumulative survival (**C**) in our study. There were no significant differences in two groups concerning the probability of HE, rebleeding and cumulative survival; the log-rank p values of which were, respectively, 0.681, 0.837 and 0.429

The median follow-up times for the schistosomiasis-induced and HBV-induced groups were 15 (2–30) and 15 (2–27) months; during the follow-up, the cumulative rate of HE is 25.0% in schistosomiasis-induced group, 22.6% in HBV-induced group, log-rank *p* = 0.681 (Fig. 3). Univariate analysis was conducted according to the occurrence of HE during the follow-up, and patients with HE had significantly lower ALB, higher Child–Pugh score, larger Spleen diameter and TIPS implantation into the left branch of the portal vein. In the multivariate analysis, only Spleen diameter (HR 0.738, 95% CI 0.565–0.965, *p* = 0.026) was an independent predictor of HE occurrence (Table 3).

**Table 3:** Association between HE development during follow-up after TIPS placement and demographic and clinical parameters

	HE (*n* = 19)	No HE (*n* = 63)	*p* value
Sex (male)	16 (84.2%)	48 (76.2%)	0.544
Age (years)	53.3 ± 10.5	51.2 ± 11.7	0.490
Confirmed schistosoma patients	5 (26.3%)	15 (23.8%)	1.000
History of hypertension and(or) diabetes	4 (21.1%)	13 (20.6%)	1.000
History of hepatic carcinoma	3 (15.8%)	4 (6.3%)	0.344
History of splenectomy	6 (31.6%)	15 (21.8%)	0.553
Laboratory parameters			
Total bilirubin (µmol/L)	24.2 ± 18.0	28.3 ± 44.8	0.710
Albumin (g/L)	28.3 ± 4.1	31.6 ± 5.9	**0.032**
Alanine aminotransferase (U/L)	31.7 ± 21.8	38.5 ± 38.6	0.480
Aspartate aminotransferase (U/L)	38.4 ± 18.0	48.0 ± 39.9	0.328
Creatinine (µmol/L)	116.4 ± 210.2	71.4 ± 38.1	0.378
Blood urea nitrogen (mmol/L)	6.1 ± 2.2	6.3 ± 3.2	0.723
Prothrombin time (s)	17.8 ± 2.8	16.8 ± 3.0	0.225
International normalized ratio	1.49 ± 0.30	1.39 ± 0.33	0.244
Hemoglobin (g/L)	79.2 ± 17.9	77.1 ± 15.8	0.625
Platelet count (10^9^/L)	106.3 ± 88.0	97.7 ± 92.5	0.723
Serum Na (mmol/L)	139.3 ± 3.1	139.0 ± 4.3	0.748
Child–Pugh score	8.4 ± 1.5	7.5 ± 1.5	**0.036**
MELD score	13.1 ± 4.5	11.4 ± 3.9	0.134
MELD-Na score	13.4 ± 4.5	12.1 ± 4.6	0.306
Imaging evaluation			
Portal vein diameter (mm)	14.6 ± 2.8	16.0 ± 4.2	0.193
Gastric coronary vein diameter (mm)	6.1 ± 2.4	6.7 ± 3.0	0.497
Splenic vein diameter (mm)	10.5 ± 3.4	12.3 ± 2.9	0.067
Spleen diameter (cm)	14.8 ± 2.2	16.9 ± 3.1	**0.023**
PVT score	1.0 ± 1.1	0.8 ± 1.1	0.582
	27.5 ± 4.0	27.4 ± 4.8	0.975
Pre-TIPS PPG (mmHg)	17.4 ± 4.0	17.1 ± 4.1	0.794
ΔPPG (mmHg)	63.3 ± 9.3	62.2 ± 8.2	0.651
Descending ratio of PPG (%)	11 (57.9%)	35 (55.6%)	1.000
Dilated balloon> 6 mm	10 (52.6%)	41 (65.1%)	0.420
Gastric coronary veins embolization	8 (42.1%)	45 (71.4%)	**0.028**
TIPS implantation into the left branch of the portal vein time in hospital (days)	11.8 ± 3.6	13.0 ± 5.2	0.388

	HR	95% CI	*p* value
Multivariate analysis			
ALB (g/L)	–	–	–
Child–Pugh score	–	–	–
Spleen diameter (cm)	0.738	0.565–0.965	0.026
TIPS implantation into the left branch of the portal vein	–	–	–

Bold values indicate statistical significance

#### Variceal Rebleeding (or First Variceal Hemorrhage)

During the follow-up, the variceal rebleeding rate was 15.0% (3 of 20 patients) and 14.5% (9 of 62 patients) in schistosomiasis-induced group and HBV-induced group, log-rank *p* = 0.837 (Fig. 3). Univariate analysis was conducted according to the occurrence of variceal rebleeding (or first variceal hemorrhage), and no significant difference was found.

#### Survival

During the follow-up, 3/20 (15.0%) patients died in schistosomiasis-induced group, one died of renal failure and the others died of variceal rebleeding. In HBV-induced group, 6/62 (9.7%) patients died, the causes of death were liver failure (*n* = 2), variceal rebleeding (*n* = 3) and unknown (*n* = 1), respectively. The cumulative survival rates between the two groups estimated with Kaplan–Meier analysis were not significantly different (log-rank *p* = 0.429) (Fig. 3). For the time-to-event analysis, the following variables including age, Child–Pugh score, MELD score and pre-TIPS portal pressure were associated closely with survival. According to the Cox proportional hazard model, the independent predictors of death were age (HR 1.083, 95% CI 1.012–1.159, *p* = 0.021) and pre-TIPS portal pressure (HR 1.159, 95% CI 1.021–1.315, *p* = 0.022) (Table 4).

**Table 4:** Predictors of cumulative survival after TIPS placement in the time-to-event analysis

	Dead (*n* = 9)	Alive (*n* = 73)	*p* value
Sex (male)	8 (88.9%)	56 (76.7%)	0.676
Age (years)	59.7 ± 13.5	50.7 ± 10.8	**0.025**
Confirmed schistosoma patients	3 (33.3%)	17 (23.3%)	0.681
History of hypertension and(or) diabetes	2 (22.2%)	15 (20.5%)	1.000
History of hepatic carcinoma	2 (22.2%)	5 (6.8%)	0.168
History of splenectomy	2 (22.2%)	19 (26.0%)	1.000
Laboratory parameters			
Total bilirubin (µmol/L)	57.1 ± 99.6	23.7 ± 24.9	0.375
Albumin (g/L)	27.7 ± 5.7	31.2 ± 5.6	0.102
Alanine aminotransferase (U/L)	31.9 ± 26.2	37.5 ± 36.3	0.673
Aspartate aminotransferase (U/L)	44.0 ± 19.3	45.9 ± 37.6	0.887
Creatinine (µmol/L)	104.4 ± 88.6	79.5 ± 110.1	0.541
Blood urea nitrogen (mmol/L)	8.18 ± 5.89	6.05 ± 2.39	0.343
Prothrombin time (s)	18.2 ± 4.4	16.9 ± 2.8	0.442
International normalized ratio	1.53 ± 0.47	1.40 ± 0.30	0.472
Hemoglobin (g/L)	74.4 ± 16.7	78.0 ± 16.2	0.554
Platelet count (10^9^/L)	60.0 ± 41.8	104.3 ± 94.0	0.193
Serum Na (mmol/L)	140.1 ± 1.9	138.9 ± 4.2	0.406
Child–Pugh score	8.8 ± 2.0	7.6 ± 1.5	**0.047**
MELD score	14.9 ± 5.0	11.5 ± 3.9	**0.025**
MELD-Na score	14.9 ± 5.0	12.1 ± 4.5	0.113
Imaging evaluation			
Portal vein diameter (mm)	15.6 ± 4.1	15.7 ± 4.0	0.927
Gastric coronary vein diameter (mm)	7.0 ± 2.9	6.5 ± 2.9	0.611
Splenic vein diameter (mm)	12.3 ± 3.4	11.9 ± 3.1	0.735
Spleen diameter (cm)	16.0 ± 3.0	16.5 ± 3.0	0.683
PVT score	1.0 ± 1.0	0.9 ± 1.1	0.725
Pre-TIPS PPG (mmHg)	28.1 ± 5.1	27.3 ± 4.5	0.615
Pre-TIPS portal pressure (mmHg)	37.4 ± 6.6	33.6 ± 4.8	**0.038**
ΔPPG (mmHg)	19.1 ± 4.5	16.9 ± 4.0	0.143
Descending ratio of PPG (%)	67.3 ± 6.8	61.8 ± 8.4	0.065
Dilated balloon> 6 mm	7 (77.8%)	39 (53.4%)	0.286
Gastric coronary veins embolization	6 (66.7%)	45 (61.6%)	1.000
TIPS implantation into the left branch of the portal vein	8 (88.9%)	45 (61.6%)	0.149
Time in hospital (days)	13.3 ± 6.4	12.6 ± 4.7	0.680
Hepatic encephalopathy after TIPS	2 (22.2%)	17 (23.3%)	1.000

	HR	95% CI	*p* value
Multivariate analysis			
Age (years)	1.083	1.012–1.159	0.021
Child–Pugh score	–	–	–
MELD score	–	–	–
Pre-TIPS portal pressure (mmHg)	1.159	1.021–1.315	0.022

Bold values indicate statistical significance

## Discussion

Schistosomiasis-induced liver fibrosis is characterized by periportal or Symmers’ pipestem fibrosis of the liver caused by deposition of eggs [28]. In *S. japonicum* infection, schistosome eggs deposited into the mesenteric venous and may be carried by blood flow into the portal circulation where they lodge in the small portal vein tributaries. These eggs are calcified and produce granulomas and fibrosis after they die, which will further result in portal hypertension, splenomegaly, gastroesophageal varices and so on [29].

For all the patients in advanced stage, full dose of antiparasitic praziquantel therapy is needed [30]. As to those with variceal bleeding, the main treatments currently are endoscopic varices ligation (EVL), endoscopic sclerotherapy (EST), devascularization surgery and splenectomy [31–33]. For patients with refractory ascites and recurrent gastroesophageal variceal bleeding, however, TIPS may be more appropriate although existing guidelines on TIPS indication do not consider this procedure as a therapeutic option for schistosomiasis-related portal hypertension.

We applied TIPS procedure on 20 patients with schistosomiasis-induced liver fibrosis and 62 patients with HBV-induced cirrhosis and then measured the portal pressure gradient (PPG) data. As the AASLD criteria suggested, hepatic venous pressure gradient (HVPG) is a measure of sinusoidal pressure and does not provide useful data in prehepatic or presinusoidal portal hypertension such as schistosomiasis-related portal hypertension [10]. Therefore, it is more reasonable to use PPG as an indicator concerning the patients in our study. The PPG value baseline of schistosomiasis-induced group is 26.4 ± 4.9 mmHg, 60.0% of whom underwent splenectomy with a still 24.8 ± 3.3 mmHg PPG baseline, which independently predicts a poor survival outcome [34]. In our results, TIPS insertion was effective in reducing the PPG values up to at least 20% from baseline in all patients, and 18 (90.0%) in schistosomiasis-induced group even dropped below 12 mmHg. There exists no difference between two groups in terms of the extent of pressure decrease; therefore, we have every reason to believe TIPS rather than splenectomy exerts prominent effect on controlling schistosomiasis-related portal hypertension.

In comparison with TIPS, splenectomy is disadvantaged in not only failing to alleviate the portal hypertension but also having additional complications such as ascites or variceal hemorrhage. We conducted univariate and multivariate analysis on patients with or without PVT, leading to the conclusion that splenectomy (HR 19, 95% CI 4–90, *p* < 0.001) was identified as independent predictors of PVT. The risk of PVT is 19 times higher in patients that underwent splenectomy than in untreated patients, suggesting the classical splenectomy since before may not be the optimal treatment for schistosomiasis-related portal hypertension.

As for the TIPS procedure in patients with PVT, there do exist certain technical difficulties, but we have accumulated rich experience upon TIPS implantation. It can be inserted as long as the inflow tract (unobstructed SMV) is intact. For patients with portal vein occlusion, portal vein recanalization and transjugular intrahepatic portosystemic shunt (PVR-TIPS) should be considered.

Some investigators have argued that TIPS should not be used in schistosomiasis because the risk of HE after TIPS insertion is higher in patients with schistosomiasis than in those with cirrhosis [12]. However, our study says otherwise. In our data, the incidence rate of HE after TIPS insertion in schistosomiasis- and HBV-induced cirrhosis is, respectively, 25.0% and 22.6% during the follow-up, *p* = 0.521, with no statistical difference.

After TIPS placement, the prognosis of PVT condition is similar in both groups; the cumulative rate of variceal rebleeding (or first variceal hemorrhage) (15.0% vs. 14.5%, *p* = 0.605) and survival (85.0% vs. 90.3%, *p* = 0.681) is also parallel. Furthermore, the independent predictors of death were age (HR 1.083, 95% CI 1.012–1.159, *p* = 0.021) and pre-TIPS portal pressure (HR 1.159, 95% CI 1.021–1.315, *p* = 0.022).

TIPS is recommended by guidelines for patients with refractory ascites and recurrent gastroesophageal variceal bleeding of HBV-induced cirrhosis [10, 35, 36]. In our study, there is no significant difference found between the two groups concerning the therapeutic outcome of TIPS insertion. Consequently, TIPS placement can be considered as a safe and effective treatment in patients with schistosomiasis-induced portal hypertension and relevant severe complications.

TIPS placement showed distinctive advantage with regard to schistosomiasis-induced variceal bleeding and refractory ascites not manageable with routine NSBB + EVL in our study. TIPS overall alleviates portal hypertension fundamentally with rare complications, despite the possible complications of HE. The incidence of HE after TIPS insertion, however, is the same as HBV-induced cirrhosis, and as long as preventive measures are taken timely after the procedure, this will not strike as a complicated problem.

A limitation of the study is the scarcity of sample quantity. For this reason, it is necessary that they were confirmed further with a higher number of patients and other treatments as control. And the other limitation is that Fluency-covered rather than Viatorr-covered stents were used because only the former was available in China. But one prospective study has shown that the long-term safety, technical success and patency of TIPS are satisfying following the Fluency-covered stents insertion [37]. Also, no significant statistical difference was found between bare metal stent/covered stent combination and Viatorr-covered stent concerning primary patency rates, survival rates and incidence of HE [38]. Thus, we do not reckon this would have much impact on the results.

## Conclusion

Our study confirms that TIPS placement is well-founded to be considered as a safe and effective treatment in patients with schistosomiasis-induced portal hypertension and relevant severe complications. We also found the risk of PVT is 19 times higher in patients that underwent splenectomy than in untreated patients.
